# In Search of the Molecular Mechanisms Mediating the Inhibitory Effect of the GnRH Antagonist Degarelix on Human Prostate Cell Growth

**DOI:** 10.1371/journal.pone.0120670

**Published:** 2015-03-26

**Authors:** Monica Sakai, Daniel B. Martinez-Arguelles, Nathan H. Patterson, Pierre Chaurand, Vassilios Papadopoulos

**Affiliations:** 1 The Research Institute of the McGill University Health Center, Montréal, Québec, Canada; 2 Department of Medicine, McGill University, Montréal, Québec, Canada; 3 Department of Chemistry, University of Montreal, Montréal, Québec, Canada; 4 Departments of Biochemistry, McGill University, Montréal, Québec, Canada; 5 Department of Pharmacology and Therapeutics, McGill University, Montréal, Québec, Canada; University of Kentucky College of Medicine, UNITED STATES

## Abstract

Degarelix is a gonadrotropin-releasing hormone (GnRH) receptor (GnRHR) antagonist used in patients with prostate cancer who need androgen deprivation therapy. GnRHRs have been found in extra-pituitary tissues, including prostate, which may be affected by the GnRH and GnRH analogues used in therapy. The direct effect of degarelix on human prostate cell growth was evaluated. Normal prostate myofibroblast WPMY-1 and epithelial WPE1-NA22 cells, benign prostatic hyperplasia (BPH)-1 cells, androgen-independent PC-3 and androgen-dependent LNCaP prostate cancer cells, as well as VCaP cells derived from a patient with castration-resistant prostate cancer were used. Discriminatory protein and lipid fingerprints of normal, hyperplastic, and cancer cells were generated by matrix-assisted laser desorption/ionization (MALDI) mass spectrometry (MS). The investigated cell lines express *GNRHR1* and *GNRHR2* and their endogenous ligands. Degarelix treatment reduced cell viability in all prostate cell lines tested, with the exception of the PC-3 cells; this can be attributed to increased apoptosis, as indicated by increased caspase 3/7, 8 and 9 levels. WPE1-NA22, BPH-1, LNCaP, and VCaP cell viability was not affected by treatment with the GnRH agonists leuprolide and goserelin. Using MALDI MS, we detected changes in m/z signals that were robust enough to create a complete discriminatory profile induced by degarelix. Transcriptomic analysis of BPH-1 cells provided a global map of genes affected by degarelix and indicated that the biological processes affected were related to cell growth, G-coupled receptors, the mitogen-activated protein kinase (MAPK) pathway, angiogenesis and cell adhesion. Taken together, these data demonstrate that (i) the GnRH antagonist degarelix exerts a direct effect on prostate cell growth through apoptosis; (ii) MALDI MS analysis provided a basis to fingerprint degarelix-treated prostate cells; and (iii) the clusters of genes affected by degarelix suggest that this compound, in addition to its known use in the treatment of prostate cancer, may be efficacious in BPH.

## Introduction

Gonadotropin-releasing hormone (GnRH) antagonists are a new class of pharmacological treatment with many potential applications [1–4]. They are currently approved to treat and manage prostate cancer (PCa) that requires androgen deprivation therapy (ADT). Low or castrated levels of circulating testosterone are desirable since testosterone promotes prostate growth [1,3,5,6]. Those low levels can be induced by using GnRH antagonists or agonists.

GnRH antagonists (such as degarelix) compete with the endogenous hypothalamic ligand GnRH to bind to the GnRH receptor (GnRHR). In men, this blockage leads to a decrease in both luteinizing hormone (LH) and follicle-stimulating hormone (FSH) release from the pituitary, and subsequently testosterone production from testes is suppressed. GnRH antagonists will act promptly in the hypothalamus—pituitary—gonadal (HPG) axis, blocking steroid synthesis. Meanwhile, before inducing low testosterone levels, GnRH agonists promote an initial stimulation of the HPG axis, causing an undesirable surge of testosterone that risks enhancement of steroid-dependent disease symptoms, or it may result in a clinical flare [7–11]. Antagonists indeed provide an immediate onset of action; in addition, no testosterone levels surge and efficient action can be reversed or sustained upon repeated dosing [4,12].

Degarelix is a synthetic decapeptide-inhibiting GnRH receptor located in the pituitary. Clinical data available on the therapeutic application of degarelix and other antagonists broadened the perspective for its use not only for PCa patients, but also for the treatment of symptomatic benign prostate hyperplasia (BPH) [13–17]. These studies using GnRH antagonists showed significant improvement of lower urinary tract symptoms (LUTS) in patients with BPH; specifically, they exhibited changes in the International Prostate Symptom Score (IPSS) and urinary flow (Qmax) [18]. Moreover, degarelix induced relief of LUTS in patients with PCa, and this improvement was more effective and occurred over a longer period in a higher percentage of patients than goserelin, a GnRH agonist [11,17,19]. LUTS is somehow considered unspecific because of its diverse etiopathology, but a reduction in prostate volume is still a possible, and there is reasonable cause for the observed relief, especially in the case of PCa and BPH patients.

Although it is unclear how GnRH agonists or antagonists suppress testosterone levels transiently (1 week or less), LUTS relief is long lasting (12–28 weeks). Many studies already proved that prostate growth is dependent on steroids; but this indirect mechanism of GnRH analogues might not be the sole reason for the observed improvement. Alternative mechanisms of action have been proposed, and an interest over the role of GnRH and GnRHR in extra-pituitary tissues (and in prostate tissues) has being raised.

GnRHR are found outside the pituitary in a variety of human tissues such as the ovaries, endometrium, placenta, breast, and prostate [20–23]. It is suggested that GnRH and its receptors could be involved in a paracrine/autocrine regulation, since ligands and receptors co-exist in normal and cancerous tissues [24]. Indeed, GnRHR is presently expressed in peripheral tissues, and it is also a binding site for GnRH analogues [25–29]; thus, these organs and cells could be a direct target for the non-central action of synthetic GnRH analogues. *In vitro* studies on the mechanisms of GnRHR showed that there are differences between pituitary cells and other cell types regarding intracellular signaling [22,23,28,30,31]. *In vivo* evidence of the non-pituitary-mediated effect on prostate is demonstrated by the inhibition of the growth of androgen-independent PCa xenografts (DU145 cells) in nude mice treated with a GnRH agonist, Zoladex [20]. In fact, some other agonists and antagonists were also shown to have an effect on prostate cells [32–34].

These diverse studies reveal the possibility that extra-pituitary tissues can be affected by GnRH analogues, and they offer a basis for direct mechanisms; however, to our knowledge, there is no investigation using degarelix specifically on prostate cells. Other reasons for the specific interest in degarelix is due the fact that this drug is currently approved to treat patients with PCa that need ADT, offering faster and more effective blockage, as well as similar cost-effectiveness and better tolerance compared to GnRH agonists [7,8,35,36]. Patients receiving degarelix over the past 5 years have tolerated the treatment well, and they have exhibited significant improvement in terms of prostate-specific antigen (PSA) levels; moreover, there is a progression-free survival (PFS) benefit for degarelix over leuprolide (including after cross-over from leuprolide to degarelix) [37]. A recent study showed that PCa patients treated with degarelix presented significant improvement in levels of PSA, PFS, and overall survival when compared to patients treated with goserelin (agonist) [38].

It is likely that the extra-pituitary GnRHR is quite versatile, and that prostate tissues are a direct target for the GnRH antagonists. Clinical investigations with GnRH antagonists and prostrate diseases are progressively showing favorable and encouraging results, but non-clinical investigations are still necessary to develop a complete comprehension of the mechanism of action. BPH and PCa patients share a common goal, which is to slow down prostate cell growth and improve LUTS. We want to evaluate the direct effect of degarelix on prostate cell growth, looking specifically for a common biological response and the pathways affected that could have an impact in the context of prostate diseases. These insights could shed light onto the molecular control of GnRHR in prostate, triggered by the use of a synthetic GnRH antagonist, degarelix.

This present work showed that prostate cell lines express both *GNRH* and *GNRHR*. Degarelix-treated (but not goserelin- or leuprolide-treated) prostate cells have decreased cell viability, which was attributed to decreased proliferation and increased apoptosis. A gene array and matrix-assisted laser desorption/ionization (MALDI) mass spectrometry (MS) analysis of prostate cells provided a global map of the genes, proteins, and lipids that are affected following degarelix exposure. The biological processes affected were not only related to cell growth, G-coupled protein receptors, and the mitogen-activated protein kinase (MAPK) pathway, but intriguingly to angiogenesis and cell adhesion as well. Changes found through MALDI analysis, as well as the profiling of proteins and lipids, provided a basis to fingerprint degarelix-treated cells.

## Material and Methods

### Cell lines

This study was conducted using six different established prostate human cell lines. Normal epithelial prostate cells (WPE1-NA22) were cultured in keratinocyte-SFM supplemented with 0.05 mg/mL of bovine pituitary extract and 5 ng/mL of epidermal growth factor (EGF). Normal stromal prostate cells (WPMY-1) were cultured in Dulbecco’s Modified Eagle’s Medium (DMEM) + 5% fetal bovine serum (FBS). Prostate benign hyperplasia cells (BPH-1 cells) were cultured in Roswell Park Memorial Institute (RPMI) 1640 media supplemented with 5% FBS. LNCaP androgen-dependent PCa cells were cultured in RPMI 1640 + 10% FBS. VCaP cells derived from a patient with hormone-refractory PCa were cultured in DMEM + 10% FBS. PC-3 androgen-independent PCa cells were cultured in F12K media + 10% FBS. The BPH-1 cell line, a generous gift from Dr. Simon Hayward, is a well-established and characterized cell line [39]. The other prostate cell lines were directly obtained from the ATCC (American Type Culture Collection, Manassas, VA, USA) under the following catalog numbers: ATCC CRL-2849 (WPE1-NA22 cells), ATCC CRL-2854 (WPMY-1 cells), ATCC CRL-1435 (PC-3 cells), ATCC CRL-1740 (LNCaP clone FGC cells) and ATCC CRL-2876 (VCaP cells).—. They were cultivated following the supplier’s recommendations. All cells were kept in 5% CO_2_ and at 37°C; they were passaged approximately at 80% confluence and the media was replaced every 2 or 3 days. All media and supplements were provided by Invitrogen (Thermo Fisher Scientific, Waltham, MA, USA).

### Quantitative RT-polymerase chain reaction (Q-PCR)

Specific gene expression in WPE1-NA22, WPMY-1, BPH-1, LNCaP, and PC-3 cells was measured by Q-PCR. Cell pellets were snap-frozen, and the total messenger RNA (mRNA) was extracted using the RNeasy Plus kit (Qiagen) that included an in-column DNAase step. Complementary DNA (cDNA) was prepared from 1 μg of total RNA using the QuantiTect Reverse Transcription Kit (Qiagen), according to the manufacturer’s instructions. Q-PCR was carried out on an LC480 machine (Hoffman-La Roche Ltd., Basel, Switzerland). Multiplex Q-PCR mix consisted of 10 μL of TaqMan Universal PCR Master Mix (Applied Biosystems, Thermo Fisher Scientific), 2 μL of cDNA, 0.7 μL of VIC-labeled *GAPDH* for cell lines, and 1 μL of FAM-labeled gene target TaqMan probes in a final volume of 20 μL. A SYBRG Q-PCR mix was used for *GNRHR1* and consisted on 10 μL of SYBRG Master Mix (Hoffman-La Roche), 2 μL of cDNA, and 1 μL of 0.4 μM primer in a final volume of 20 μL. VIC-labeled *GAPDH* was used as an endogenous control to normalize the FAM-labeled targets obtained in cell lines. Results are presented as a ratio between the target gene relative to and the reference gene normalized to the levels of an expressing control. S1 and S2 Tables list the TacMan probes and primer sequences used for measuring the gene expression levels.

### Immunoblot analysis

Cells were lysed in RIPA buffer 1X [20mM Tris-HCl (pH 7.5), 150mM NaCl, 1mM Na_2_EDTA, 1mM EGTA, 1% NP-40, 1% sodium deoxycholate, 2.5mM sodium pyrophosphate, 1mM β-glycerophosphate, 1mM Na_3_VO_4_, 1μg/ml leupeptin] (Cell Signaling, Beverly, MA, USA) supplemented with protease inhibitor cocktail (cOmplete; Roche Diagnostics, Indianapolis, IN, USA). Protein levels were measured by the Bradford method using the Bio-Rad Protein Assay kit (Bio-Rad Laboratories, Hercules, CA, USA), with Bovine Serum Albumin as a standard.

Protein extracts (40–50μg) were solubilized in sample buffer [30 mM Tris—HCl (pH 6.8), 2% SDS, 40mM DTT, 1 mM EDTA, 4% glycerol, and 0.01% bromophenol blue], heated for 5 min at 95°C, loaded onto a 4–20% (GnRHR immunoblots) or 10–20% (Caspase immunoblots) Tris—Glycine gels (Invitrogen, Carlsbad, CA, USA), and transferred to nitrocellulose membranes. Non-specific staining was blocked with 10% non-fat milk in TTBS [20mM Tris—HCl (pH 7.5), 0.5 M NaCl, and 0.04% Tween 20]. Membranes were incubated with primary antibodies overnight at 4°C. The primary antibodies used (dilution and catalog numbers) were anti-GnRHR (1:100, MA5-11538, Thermo Fisher Scientific), and anti-cleaved Caspase-3 (1:1000, CS9661), anti-Caspase-7 (1:1000, CS 9494), anti-cleaved Caspase 9 (1:1000, CS 9501) obtained from Cell Signaling.

Specific protein bands were detected using the Immobilon Western kit (Millipore Corporation, Billerica, MA, USA), and images were captured using the ImageQuant LAS4000 imaging system (FujiFilm & GE Healthcare Life Sciences, Baie d’Urfe, QC, Canada). Equal protein loading was verified by re-probing the blots with anti-β-actin (1:1000, CS4967, Cell Signaling). Densitometric analysis of the immunoreactive protein bands was performed using Multi-Gauge Software version 3.1 (FujiFilm Corporation Life Sciences, Mississauga, ONT, Canada).

### Cell culture treatments

Cell lines were seeded at a density of 2–3×10^3^ cells/well/100 μL in a 96-well cell culture plate, with the exception of VCaP, which was seeded at 2×10^4^ cells/well/100 μL.

Cells were allowed to attach for 24–48 hours (h). After that, they were treated with different concentrations of degarelix, a GnRH antagonist (0.1 to 10 μM); in its pure peptide form (FE200486) kindly provided by Ferring Pharmaceuticals (Saint-Prex, Switzerland). Leuprolide acetate and goserelin acetate (GnRH agonists) were used as well (1 nM to 100 μM) from Sigma-Aldrich Co. (St. Louis, MO, USA). All of them were diluted in distilled water that was then used as a control in an appropriate concentration.

Cell viability (MTT assay) was accessed at different time points (24, 48, and 72h) after degarelix, leuprolide or goserelin individual treatments. Caspase studies were performed on prostate cells treated with degarelix. Caspase 3/7 activation was accessed at different time points after treatment (24, 48, and 72h), caspase 8 and 9 activation were accessed specifically at 48h in BPH-1 cells and 72h in LNCaP cells.

For experiments where treatments consisted of a combination of degarelix and leuprolide, BPH-1 cells were allowed to attach for 24h. Cells were either pre-treated with leuprolide (10μM) one hour prior to the addition of various concentrations (0.001 to 10 μM) of degarelix or they were pre-treated with degarelix (10μM) one hour prior to the addition of various concentrations (0.001 to 100 μM) of leuprolide. Cell viability was accessed at (24, 48, and 72h after last treatment.

### MTT cell viability assay

The kit used was the Cell Proliferation Kit I (MTT) from Hoffman-La Roche Ltd. Briefly, 10 μL of MTT labeling reagent was added to each well and the plates were incubated at 37°C for 4h. Then, 100 μL of solubilization solution was added, and the plates were incubated at 37°C, overnight, in a humidified atmosphere. The final reaction was measured using the Victor automated plate reader at 550–600 nm. The obtained absorbance directly correlates to the number of live and metabolically active cells, providing an indication of cell viability.

### Caspase 3/7, Caspase 8, Caspase 9 activation

The kit used was the ApoTox-Glo Assay from Promega Corporation (Fitchburg, WI, USA). This assay measures caspase activation 3/7 within a single assay well using the Caspase-Glo Assay Technology. The luminogenic caspase 3/7 substrate is added (100 μL) and plates are incubated at room temperature for 30–60 minutes. The caspase cleavage of the substrate generates a “glow-type” luminescent signal produced by luciferase. The same principle and protocol apply to the kits Caspase-Glo 8 and 9 (Promega Corporation) used to determine caspase 8 and 9 activities. Luminescence (RLU) reading is proportional to the amount of caspases 3/7, 8 and 9 activity present in each well.

### Global gene expression studies

BPH-1 cells were seeded in 100 mm dishes (2×10^5^/10 mL). Cells were allowed to attach and they were treated with degarelix (10 μM). BPH-1 cells were harvested 6 and 24h after the treatment. These time points were chosen because we wanted to identify the effect of degarelix on gene expression changes when the MTT assay started to indicate a decrease in cell viability following degarelix treatment. Cell pellets were washed in phosphate buffered saline (PBS) and all of the supernatant was removed. These dry pellets were flash-frozen in liquid nitrogen and kept in -80°C for the RNA extraction. Total mRNA was extracted using the RNeasy Plus kit (Qiagen). The total RNA was submitted to McGill University and the Genome Quebec Innovation Centre for RNA quality control and hybridization to Affymetrix U133 Plus gene chip. Quality control, normalization, and analysis of the differential expression were sent to GenexAnalysis Service and were carried out as previously described [40].

### Statistical analysis of gene expression, MTT, caspase assays and immunoblots

Statistical analysis was performed using Prism version 5.0 (GraphPad Software, Inc., La Jolla, CA, USA). Data were analyzed using Student’s *t* test and one- or two-way analysis of variance (ANOVA), followed by post-hoc Tukey multiple comparison or Bonferroni tests when appropriated. Data are presented as means ± standard error, and *p*<0.05 was considered significant. Experiments were performed in triplicate and repeated at least three times independently. Details are displayed in the description of the results

### Matrix-assisted laser desorption/ionization (MALDI) mass spectrometry (MS) and data analysis

WPE1-NA22, BPH-1, and LNCaP cells were seeded at a density of 2–4.5×10^5^ cells/10 mL in 100 mm dishes. Cells were allowed to attach for 24–48h. After that, they were treated with degarelix, a GnRH antagonist (10 μM) in its pure peptide form, diluted in distilled water for 24h. Cells were harvested and washed in cold PBS. The supernatant was removed and the pellets were snap-frozen in liquid nitrogen. Samples were kept at -80°C in a freezer until the MALDI analysis. For each cell type and condition (control or degarelix-treated), three independent cultures were made. From each of these, three separate MALDI spots were prepared (see below). From each spot, 5–7 independent spectra were manually acquired, depending on the MS signal quality. Each spectrum was the average of 1,000 consecutive laser shots.

On the day of the analysis, cells pellets were thawed on ice and numerous 0.5 μL aliquots of the cell pellet were suspended in a solution of MALDI matrix and deposited on a 384-well MALDI target plate. For the protein analysis, 0.5 μL of a 20 mg/mL sinapinic acid matrix solution prepared in acetonitrile (ACN):trifluoroacetic acid (TFA) 0.2%– 50:50 was immediately added in the wells; then, a second spot of matrix was re-applied upon dryness. For the lipid analysis, 0.5 μL of 20 mg/mL of a 2,6-dihydroxyacetophenone matrix solution in ACN:TFA 0.2%– 50:50 was added and re-applied upon dryness.

MS measurements were performed on a Bruker Daltonics Ultrafextreme MALDI TOF/TOF mass spectrometer (Bruker Corporation, Billerica, MA, USA). Protein MS measurements were performed in the linear mode geometry under +25 keV of energy and under optimized delayed extraction conditions. MS data were collected in the 2,000–20,000 mass-to-charge (*m/z*) range. Lipid MS measurements were performed in the reflectron mode geometry under +25 keV or -20 keV of energy and under optimized delayed extraction conditions. MS data were collected in the 600–2,000 *m/z* range.

Statistical analyses of the MS data were performed using Bruker’s ClinProTools 3.0 software (Bruker Corporation). Preprocessing consisted of noise and background subtraction, total ion current intensity normalization, and MS data realignment. After preprocessing, peaks were picked using an s/n ratio of 5.0 on the average mass spectrum. The peak list matrix was then submitted for principal components analysis (PCA) within ClinProTools 3.0.

## Results

### Cell lines—MALDI MS

**Fig 1:**
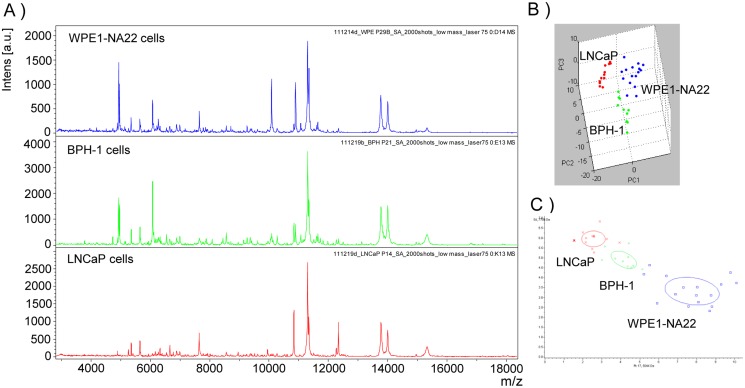
Protein spectra of human prostate cells. (A) MALDI MS protein mass spectra, (B) principal component analysis (PCA) score plot, and (C) comparison of the two most discriminant peaks across samples.

To assess the degree of molecular differentiation between the WPE1-NA22, BPH-1, and LNCaP cells, their protein composition was screened by MALDI MS. Fig. 1A presents the representative protein MS spectra for each cell type. A significant number of protein signals were observed to be differentially expressed between these three prostate cell types, and multivariate PCA clearly differentiates the cell cultures (Fig. 1B-C). Together, these results identify unique protein signatures of each cell line.

### Changes in the expression of genes important for the action of degarelix

**Fig 2:**
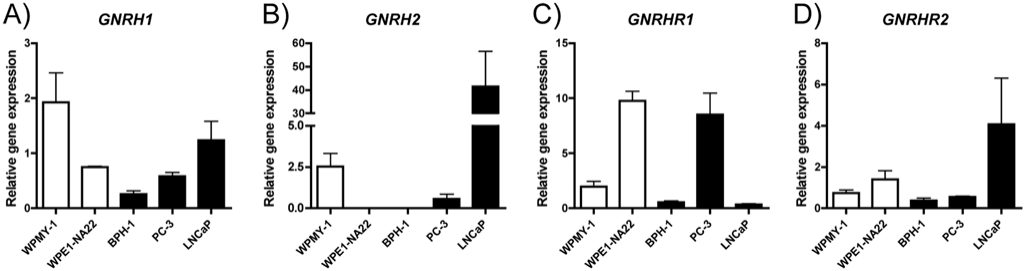
Gene expression of prostate human cell lines for *GNRH* and its receptor subtypes 1 and 2. (A) *GNRH1*, (B) *GNRHR1*, (C) *GNRH2*, and (D) *GNRHR2*. The white bars show normal cells and the black bars show hyperplasia or cancer cells. Results are presented as a ratio between the target gene relative to the reference gene normalized to the levels of the control. Results shown are means ± standard error from 3 independent experiments performed in triplicates.

Considering the interest of the work on the mechanism of action of degarelix *in vitro*, we investigated the expression of *GNRH* endogenous ligands, *GNRHR*, and their isoforms. Fig. 2A-D show that the *GNRH1* and *GNRH2* peptides and receptor mRNAs were present in the cell lines studied at different expression levels. Of interest, *GNRHR1* was present at low levels in BPH-1 cells, whereas it was expressed at high levels in WPE1-NA22 cells (Fig. 2B). *GNRHR2* is present in all cell lines as well (Fig. 2D). Experiments were performed in triplicate and repeated at least three times independently. The results are expressed as the mean ± standard error of the mean. One-way ANOVA revealed differences among *GNRH1* means (*p*<0.05) and Tukey’s multiple comparison test revealed significant difference between WPMY-1 and BPH-1 (*p*<0.05). For *GNRH2*, One-way ANOVA revealed differences among means (*p*<0.01) and Tukey’s multiple comparison test revealed significant difference between WPMY-1 vs LNCaP (*p*<0.05), WPE1-NA22 vs LNCaP (p<0.01), BPH-1 vs LNCaP (*p*<0.01) and PC-3 vs LNCaP (*p*<0.05), while all other comparison showed *p*>0.05. For *GNRHR1*, One-way ANOVA revealed differences among means (*p*<0.001) and Tukey’s multiple comparison test revealed significant difference between WPMY-1 vs WPE1-NA22 (*p*<0.01), WPMY-1 vs BC-3 (*p*<0.01), WPE1-NA22 vs BPH-1 (*p*<0.001), WPE1-NA22 vs LNCAP (*p*<0.001), BPH-1 vs PC-3 (*p*<0.01) and PC-3 vs LNCaP (*p*<0.01). For *GNRHR2*, One-way ANOVA revealed no differences among means (*p*>0.05).

### GnRHR protein levels

**Fig 3:**
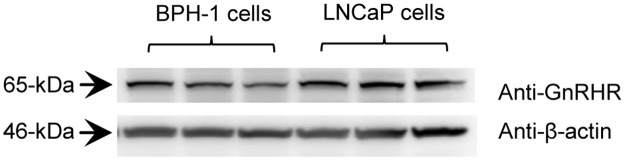
GnRHR levels in BPH-1 and LNCaP cells. Representative immunoblots of BPH-1 and LNCaP cell extracts. Each lane represents an independent cell passage (n = 3).

Immunoblot analysis of GnRHR expression showed a clear band of estimated size of 65-kDa (as expected) present in both BPH-1 and LNCaP cells (Fig. 3). Each lane shown is representative of an independent cell passage; beta actin was used as a loading control.

### Cell viability after degarelix treatment

**Fig 4:**
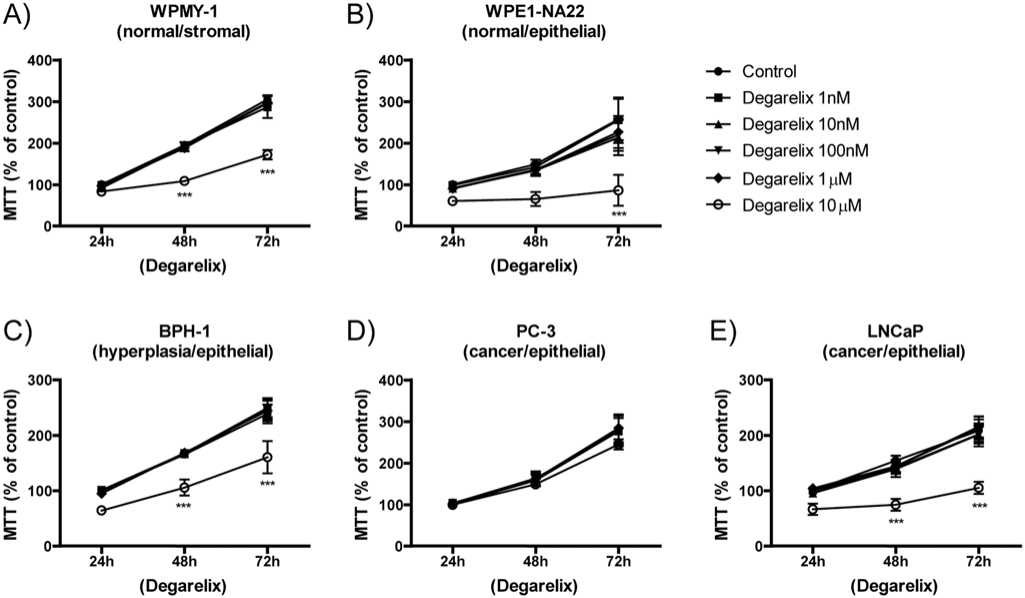
MTT assay showing the viability of prostate cell lines following treatment with the GnRH antagonist, degarelix. (A) WPMY-1, (B) WPE1-NA22, (C) BPH-1, (D) PC-3, and (E) LNCaP. Data are expressed as the percentage of the respective controls and the average ± standard error. Each assay was done in triplicate in at least 3 independent experiments for each cell line. Two-way ANOVA indicated there was a significant difference overall for degarelix treatment (p<0.001), and the posttest indicated that there were differences against each control, as displayed in each graph (***p<0.001).

In order to analyze the direct effect of degarelix (a GnRH antagonist) on prostate cell growth, cells were treated *in vitro* in a time course experiment. Experiments were performed in triplicate and repeated at least three times independently. The results are expressed as the mean ± standard error of the mean. Fig. 4A-E show the cell viability of different prostate cells after 24, 48, or 72h of treatment. Viability is decreased in four out of five of the tested cell lines (normal, hyperplasia, and cancer), with the exception of androgen-independent PC-3 cells. Two-way ANOVA revealed that treatment with degarelix and exposure time are significant sources of variation (*p*<0.0001) and post-hoc Bonferroni comparison test revealed significant differences between control and degarelix (10μM). The overall differences found for degarelix treatment include: 4A) WPMY-1 cells at 48 and 72h (*p*<0.001); 4B) WPE1-NA22 cells at 72 hours (*p*<0.001); 4C) BPH-1 cells at 48 and 72h (*p*<0.001); and 4E) LNCaP cells at 48 and 72h (*p*<0.001). When the treated groups were individually compared with controls, we observed that 10 μM of degarelix reduces cell viability at various time points, as indicated individually in each graph (***p<0.001). For PC-3 cells (Fig. 4D), Two-way ANOVA revealed that only time is a significant source of variation (*p*<0.0001) and post-hoc Bonferroni comparison test revealed no significant difference between control and degarelix at various concentrations analyzed (*p*>0.05).

### Cell viability after goserelin and leuprolide treatment

**Fig 5:**
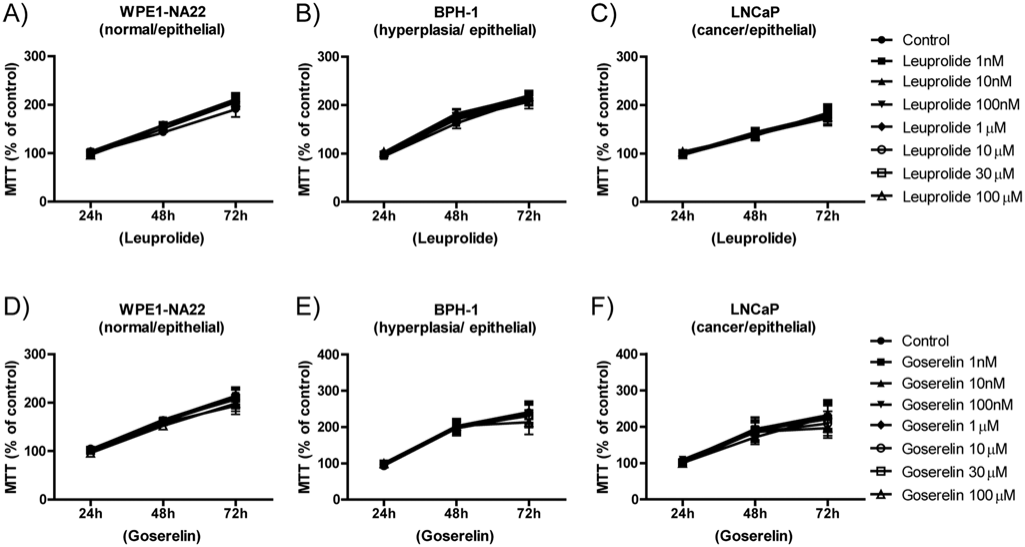
MTT assay showing the viability of WPE1-NA22, BPH-1, and LNCaP cell lines following treatment with GnRH agonists. (A-C) Leuprolide and (D-E) goserelin. Note that the different cell lines are displayed in each column. The data are expressed in terms of the percentage of the respective control and the average ± standard error. Each assay was done in triplicate with at least n = 3 independent experiments for each cell line. Two-way ANOVA displayed p>0.05; there was no difference overall for the treatments.

After treatment with the GnRH agonists leuprolide (Fig. 5A-C) or goserelin (Fig. 5D-F), cell viability remained the same in those three cell lines analyzed: WPE1-NA22, BPH-1, and LNCaP. Concentrations of the agonists were used at concentrations as high as 100 μM; however, treated cells displayed similar growth to the control cells

Specifically, for WPE1-NA22 cells (Fig. 5A and D), BPH-1 (Fig. 5B and E) and LNCaP (Fig. 5C and F), Two-way ANOVA revealed that only time was a significant source of variation (*p*<0.0001) and post-hoc Bonferroni comparison test revealed no significant difference between control and degarelix at various concentrations tested (*p*>0.05). Also, BPH-1 cells pre-treated with leuprolide (10 μM) before the addition of increasing concentrations of degarelix showed drug response in the viability curves obtained at 24, 48 and 72h (S3 Fig.) similar to that seen with degarelix alone (Fig. 4). Pre-treatment with degarelix (10 μM) followed by treatment with increasing concentrations of leuprolide did not affect the inhibitory effect of degarelix on BPH-1 cell viability over time (S3 Fig.).

### VCaP viability in response to GnRH analogue treatment

**Fig 6:**
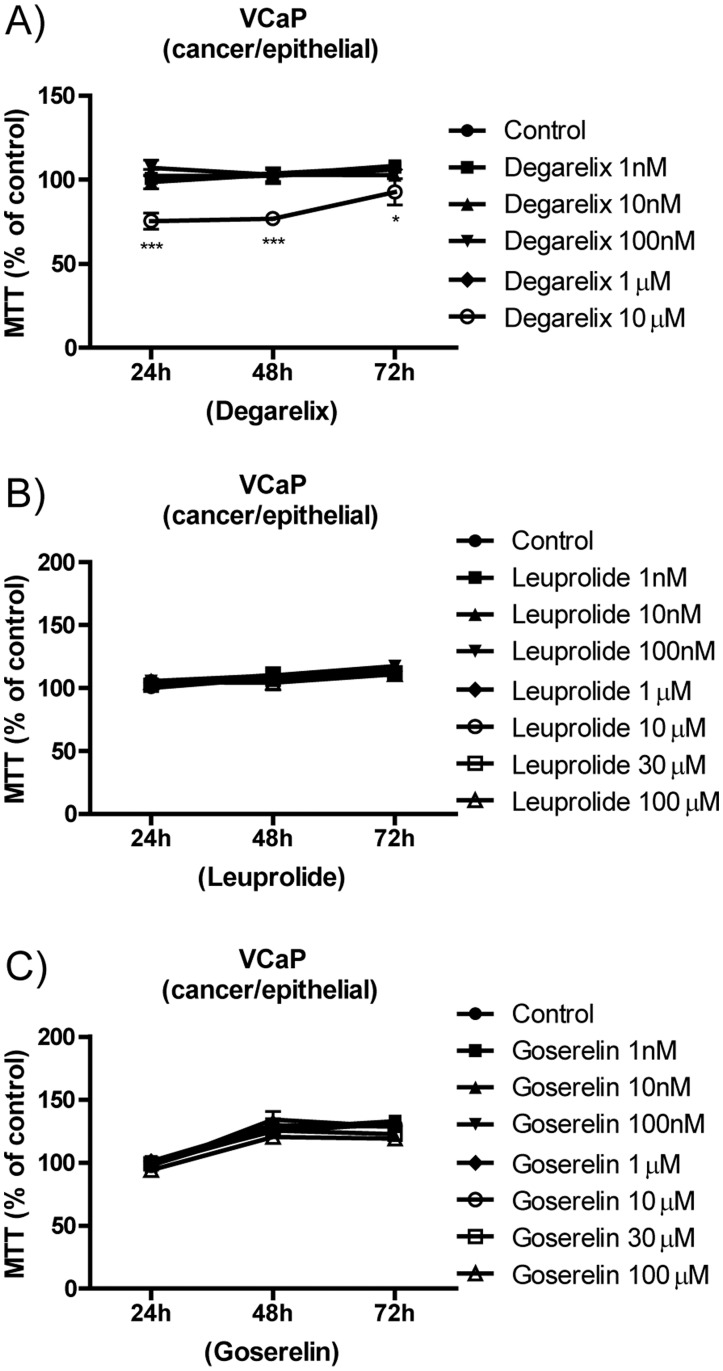
MTT assay showing the viability of the VCaP cell line after treatment with the GnRH antagonist or agonists. (A) Degarelix, (B) leuprolide, and (C) goserelin. The data are expressed in terms of the percentage of the respective control and the average ± standard error. Each assay was done in triplicate with at least n = 3 independent experiments for each cell line. Two-way ANOVA indicates that there was a significant difference overall for degarelix treatment (p<0.001), and the posttest indicated that there were differences against each control, as displayed in each graph (*p<0.05). ANOVA displayed p>0.05; for the leuprolide and goserelin groups, there was no difference overall for the treatments.

VCaP cells (derived from a patient with castration-resistant PCa) had a similar pattern of response to GnRH analogues as the other cancer cells. Degarelix-treated cells exhibited decreased viability (Fig. 6A), and cell viability remained the same after leuprolide or goserelin treatment (Fig. 6B and C). For the degarelix-treated cells (Fig. 6A), Two-way ANOVA revealed that treatment with degarelix and time are significant sources of variation (*p*<0.001 and *p*<0.01, respectively) and post-hoc Bonferroni comparison test revealed significant difference between control and degarelix 10μM at 24h and 48h (*p*<0.001) as well as at 72h (*p*<0.05). For the leuprolide- and goserelin-treated VCaP cells (Fig. 6B and C), Two-way ANOVA revealed that only time was a significant source of variation (*p*<0.001) and post-hoc Bonferroni comparison test revealed no significant difference between control and Degarelix at various concentrations tested (*p*>0.05).

### Apoptosis measurement after degarelix treatment

**Fig 7:**
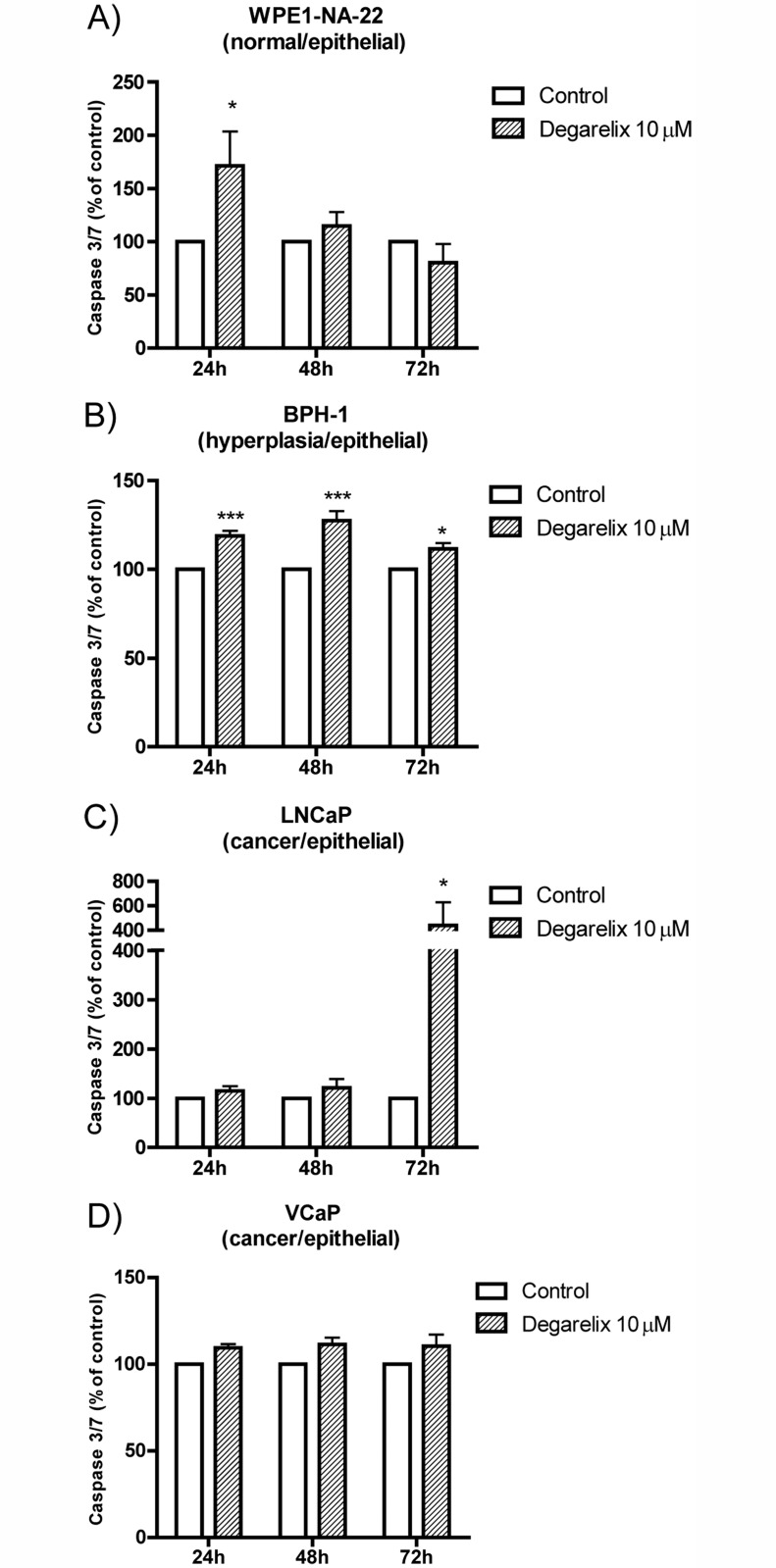
Caspase 3/7 activation levels showing apoptosis of the prostate cell lines after treatment with the GnRH antagonist, degarelix. (A) WPE1-NA22, (B) BPH-1, (C) LNCaP, and (D) VCaP. The data are expressed in terms of the percentage of the respective control and the average ± standard error. Each assay was done in triplicate with at least n = 3 independent experiments for each cell line. Two-way ANOVA indicated that there was a significant difference overall for degarelix treatment (p<0.001 for WPE1-NA22 and BPH-1; p<0.05 for VCaP), and the posttest indicated that there were differences against each control, as displayed in each graph (*p<0.05 and ***p<0.001).

Fig. 7 shows the caspase 3/7 activation measurement in four different prostate cells. Degarelix treatment induces a significant increase on caspase 3/7 activation compared to control in normal, hyperplasia, and cancer cells. The increase in this effector caspase indicates that cells are undergoing cell death (specifically apoptosis) using the caspase cascade after degarelix treatment; this could explain the decreased cell viability.

Caspase 3/7 activation statistical analysis revealed that for WPE1-NA22 cells (Fig. 7A), Two-way ANOVA revealed that only treatment with degarelix was a significant source of variation (*p*<0.001) and post-hoc Bonferroni comparison test revealed significant difference between control and degarelix 10μM at 24h (*p*<0.05). For BPH-1 cells (Fig. 7B), Two-way ANOVA revealed that treatment with degarelix and time are a significant source of variation (p<0.001 and p<0.05) and post-hoc Bonferroni comparison test revealed significant difference between control and Degarelix 10μM at 24h and 48h (*p*<0.001) and 72h (*p*<0.05). For LNCaP cells (Fig. 7C), Two-way ANOVA revealed that treatment with degarelix and time are not a significant source of variation (*p*>0.05) and post-hoc Bonferroni comparison test revealed significant difference between control and Degarelix 10μM at 72h (*p*<0.05). For VCaP cells (Fig. 7D), Two-way ANOVA revealed that treatment with degarelix was a significant source of variation (*p*<0.05) and post-hoc Bonferroni comparison test revealed significant no difference between control and Degarelix 10μM (*p*>0.05).

**Fig 8:**
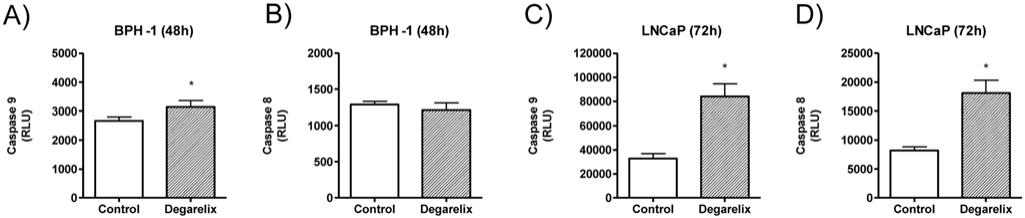
Caspase 9 (intrinsic pathway) and 8 (extrinsic pathway) activation levels in BPH-1 and LNCaP cells. (A) and (B), Control and degarelix-treated for 48h BPH-1 cells. (C) and (D), Control and degarelix-treated for 72h LNCaP cells. Results shown are means ± standard error from 3 independent experiments performed in triplicates. *, indicates p<0.05.

Fig. 8 shows the caspase 8 and 9 activation measurement in BPH-1 cells and LNCaP cells at two specific time points. For the statistical analysis, treated-groups were compared to their respective control by using the Student’s *t* test. Degarelix induced a significant increase in caspase 9 activation in BPH-1 cells (Fig. 8A, *p*<0.05), whereas caspase 8 remained unchanged (Fig. 8B, *p*>0.05). LNCaP cells presented a significant increase not only in caspase 9 (Fig. 8C, *p*<0.05) but as well in caspase 8 activation levels (Fig. 8D, *p*<0.05) after degarelix treatment. Experiments were performed in triplicate and repeated at least three times.

**Fig 9:**
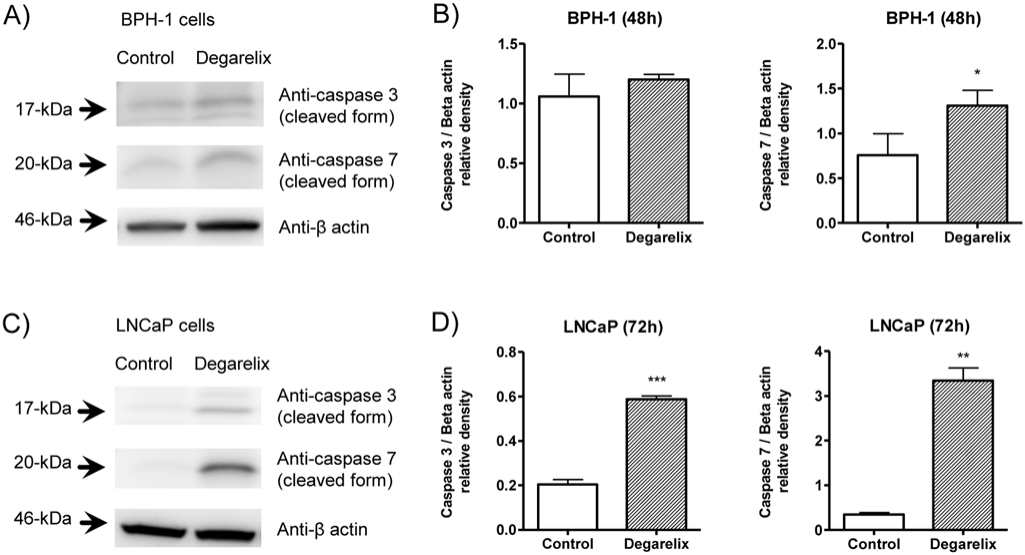
Levels of Caspases 3 and 7 in BPH-1 and LNCaP cells. (A) and (B), Representative immunoblots of BPH-1 cell extracts from control and degarelix-treated cells for 48h and their respective densitometric analysis. (C) and (D), Representative immunoblots of LNCaP cell extracts from control and degarelix-treated cells for 72h and their respective densitometric analysis. Results shown are means ± standard error from 3 independent experiments. *, **, *** indicate p<0.05, p<0.01 and p<0.001, respectively.

Immunoblot analysis of caspase 3 and 7 in their cleaved forms confirmed the results previously described for BPH-1 and LNCaP cells (Fig. 9). Fig. 9A and C illustrate representative immunoblots for caspase 3, caspase 7 and beta actin incontrol and degarelix-treated BPH-1 and LNCap cells, respectively. Fig. 9B and D shows the quantification of the immunoblots; while cleaved caspase 3 was not significantly increased, cleaved caspase 7 was found in increased levels in degarelix-treated BPH-1 cells (Fig. 9B, *p*>0.05). Interestingly both cleaved forms are increased in degarelix-treated LNCaP cells (Fig. 9D, *p*<0.001 for caspase 3 and *p*<0.01 for caspase 7). Experiments were performed in triplicate and repeated at least three times. For the statistical analysis, treated-groups were compared to their respective control using Student’s *t* test).

### MALDI MS cell analyses

The raw MS data were plotted as two-dimensional (2D) density plots or gel views for a quick visualization of the prostate cell molecular expression patterns for both proteins and lipids (S1 Fig.). Each gel view has two datasets: control and degarelix-treated. Each horizontal line represents an individual spectrum, and each vertical line represents a detected peak shown on a rainbow color scale according to its relative abundance. This visualization provides quality control of the individual sample acquisitions, as well as an immediate comparison of the many spectra, to detect changes in the spectral patterns. When comparing the protein or lipid MS profiles between control and degarelix-treated cells, a quick overview of the 2D gel views indicated that across the three cell types, WPE1-NA22 cells seems to present more contrasting differences, followed by the BPH-1 and LNCaP cells.

**Fig 10:**
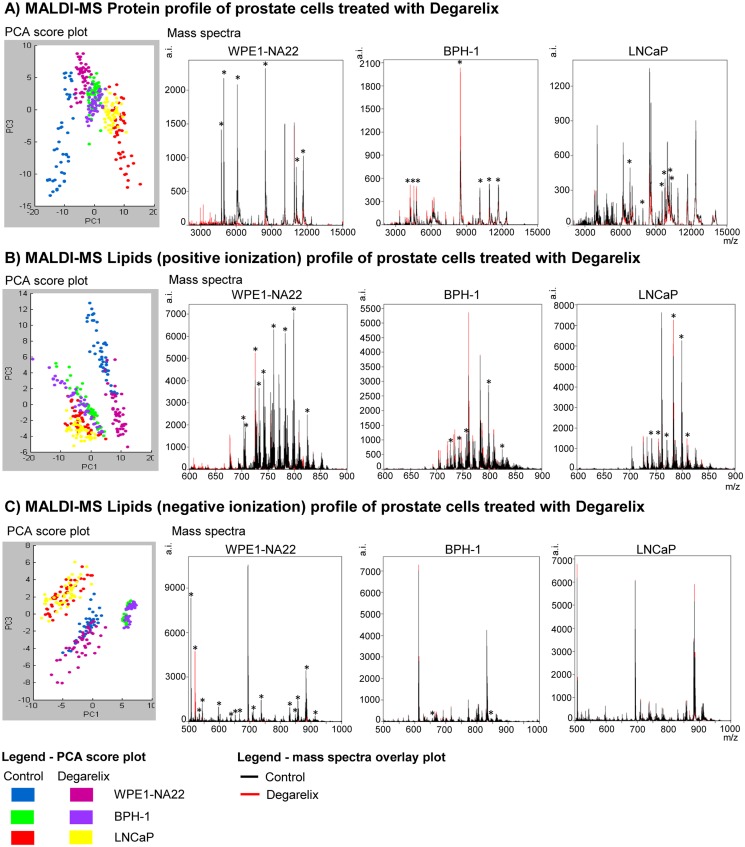
MALDI MS profiles. The profiles of prostate cells treated with degarelix for the protein (A) and lipids acquisition in positive (B) and negative polarity (C). Each acquisition shows the PCA score plots of all data originating from each sample group from the respective analyses. The average mass spectra comparisons of the control versus treatment for each sample type and acquisition type (black indicates the control, red indicates treatment, and the asterisks highlight differentially expressed signals).

Like the 2D gel views, the corresponding PCA plots showed that the WPE1-NA22 cells presented clusters that featured a more discriminant profile of proteins and lipids, followed by the BPH-1 and LNCaP cells (Fig. 10). An overlay of the sum MS spectra for each cell type illustrated some specific discriminant proteins or lipids of prostate cells (control or degarelix-treated). Some of those lower or higher m/z signal intensities are highlighted by asterisks. Degarelix was also observed at its expected mass of 1,630.75 g/mol in the different cell samples analyzed (S2 Fig.).

### Global changes in gene expression

**Fig 11:**
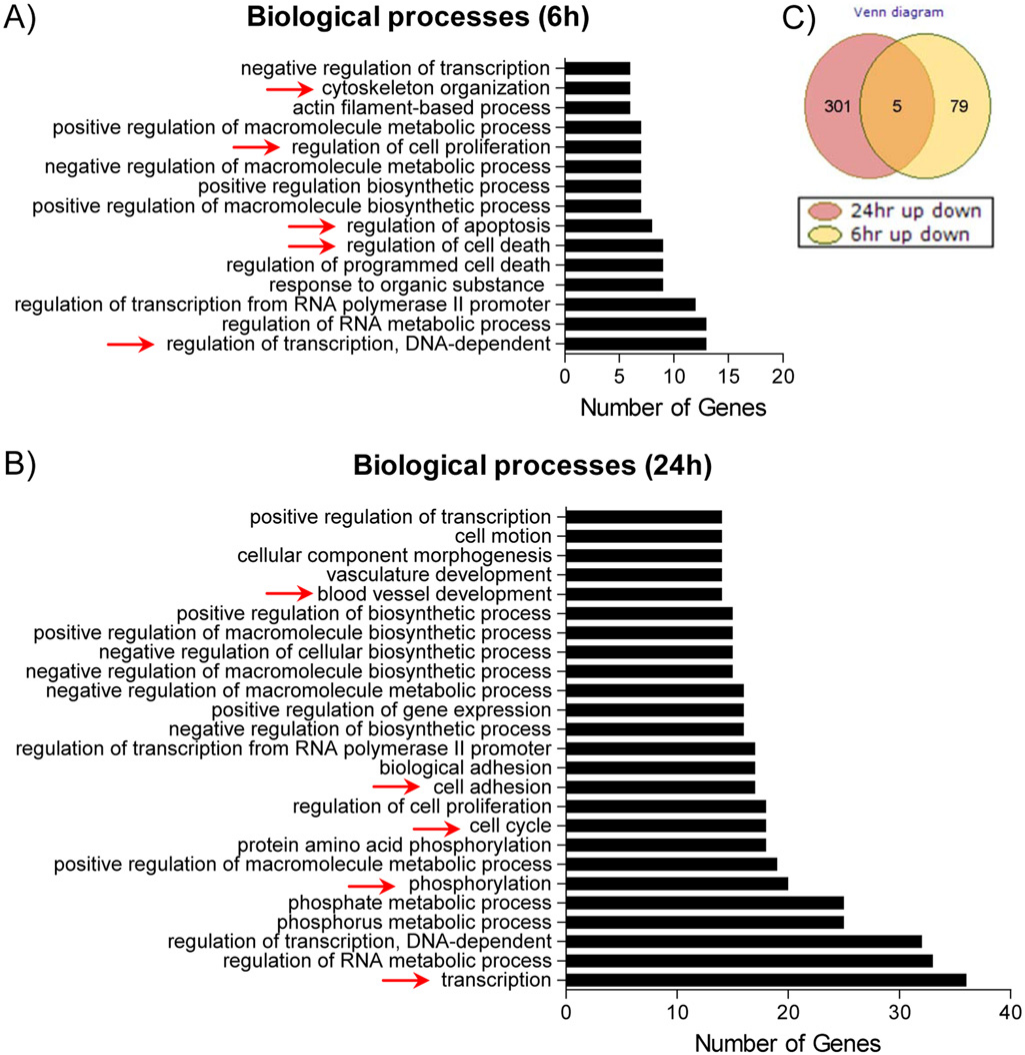
Gene ontology classification (based on biological processes) of degarelix-deregulated genes on BPH-1 cells. (A-B) The number of genes deregulated by degarelix in various biological processes after 6 and 24h, respectively. The arrows point to potential interesting processes in the BPH and degarelix context. (C) Venn diagram illustrating the number of genes deregulated by degarelix at 6 and 24h.

To investigate the early gene changes triggered by degarelix, BPH-1 cells were treated for 6 and 24h with 10 μM of degarelix. We found that at 6h of treatment, 70 genes were upregulated and 14 genes were downregulated. The data show discrete changes in gene expression that ranged between a 1.3-fold decrease and a 1.5-fold increase. A cutoff of a ±1.15-fold change was selected for further gene analysis. Results obtained at 24h showed that 185 genes were upregulated (34 genes >2-fold) and 121 genes were downregulated (28 genes >2-fold) in BPH-1 cells. Fig. 11C shows a Venn diagram that was used to compare significant gene expression changes at 6h (84 genes) and 24h (306 genes), which shows that five genes were commonly affected at both time points.

To gain further insight into the gene changes, we submitted the 84 and 306 up-/downregulated genes (from the 6 and 24h time points) to the DAVID (http://david.abcc.ncifcrf.gov/) bioinformatics website to obtain a list of the biological processes affected by degarelix (Fig. 11A and B). The arrows point toward interesting processes and the number of genes deregulated after degarelix treatment. We identified early changes (6h) in the regulation of transcription, cell death, cell apoptosis, cell proliferation, and cytoskeleton organization (Fig. 11A). At 24 hours (Fig. 11B), we identified significant changes in genes associated with transcription, phosphorylation, cell cycle, cell adhesion, and blood vessel development.

We conducted functional annotation clustering using the DAVID bioinformatics portal to identify genes with similar functions after 24h of degarelix treatment. We identified that clusters related to blood vessel development (Cluster 1), MAPK pathway (Cluster 2), apoptosis (Cluster 3), cell receptors (Cluster 4), inflammatory response (Cluster 5), and EGF-like genes (Cluster 6) were affected by degarelix. The complete list of genes associated with the clusters mentioned above can be found in the supplemental material (S3 Table).

## Discussion

Our current study shows that all of the tested human prostate cell lines express the *GNRH* peptide and its receptors. Similar results were found with prostate specimens, regardless of whether they were of normal, hyperplasia, or cancer origin (data not shown). While it has been described that extra-pituitary cells and tissues express GnRH and GnRHR [41–43], we needed to confirm these results to investigate the specific direct effects of degarelix on prostate cell lines. In other studies, GnRHR was not found in all of the prostate samples analyzed; however, they were still found in the majority of tissues and with binding sites for GnRH analogues [43]. We report herein that GnRHR in both BPH-1 and LNCaP cells, widely used in our studies.

We found decreased cell viability following degarelix treatment at different time points, as indicated by MTT assay. This indicates that degarelix acts directly on prostate cell growth, as these cells express *GNRHR*, and it affects nontumorigenic (normal epithelial and stromal cells), hyperplasia-type (BPH-1 cells), and tumorigenic cells (androgen-responsive LNCaP and castration-resistant VCaP cells). PC-3 androgen non-responsive cells are not sensitive to degarelix, but the reasons for this are unknown. It is unlikely that this could be androgen-mediated since, similar to what was observed for the PC-3 cells, WPE1-NA22 cells do not express androgen receptors (data not shown); however, they are sensitive to degarelix. It is possible that degarelix inhibits a pathway whereby PC-3 cells do not rely on growth, or which does not control growth in PC-3 cells; different prostate cell lines have variable responses to androgens and growth factors as their particular *in vitro* characteristics [44–46].

The MTT assay is very sensitive when measuring the cytotoxic effects of drugs *in vitro* as it indicates the amount of viable cells; however, the results needed to be extended with other assays, and for that we measured apoptosis. We found that degarelix treatment induces an increase of caspase 3/7 activation in all prostate cells analyzed. These data obtained using a sensitive luminescent assay were further confirmed at the protein level with BPH-1 and LNCaP cells. Indeed, BPH-1 presented a significant increase in caspase 7 (cleaved form) and LNCaP cells presented a notable increase in caspase 3 and 7 (cleaved forms) following degarelix treatment. This caspase activation can be triggered by the extrinsic death receptor pathway (which is more related to the immune system) and the intrinsic mitochondrial pathway [47–49]. To access that, activation of initiator caspases 8 and 9 were investigated. While BPH-1 cells presented an increase only in caspase 9 activity, LNCaP presented an increase in both caspases 8 and 9 activities. Caspase 9 is recognized for being involved in apoptosis through the intrinsic and caspase 8 through the extrinsic pathway. Of interest, apoptosis is the major cell death pathway involved in removing unwanted cells, and most anti-cancer therapies rely on the activation of this process [49–51].

In this respect, cetrorelix (another GnRH antagonist) is shown to have direct inhibitory effects over BPH-1 cell line growth; it decreases the expression of the proliferating cell nuclear antigen (PCNA). In this same study, cetrorelix also inhibited the stimulatory effect of growth factors IGF-I and -II, and FGF-2 [33]. It was shown that part of the underlying mechanism involves cell cycle arrest, and that cetrorelix induced changes in the transcripts of pro-inflammatory cytokines [32,52,53]. We are currently demonstrating in this work that different prostate cell lines (hyperplasia and cancer) are a direct target of another important GnRH antagonist, degarelix, through a decrease in cell viability and an increase in apoptosis. Moreover, we also show that the GnRH agonists leuprolide and goserelin do not affect prostate cell viability, even when tested at a concentration of 100 μM, indicating that the effect might be specific for the antagonists, at least in prostate cells. Also, there is no pharmacological interaction between leuprolide and degarelix (antagonism or synergism), suggesting that the effect of degarelix on prostate cells are specific to the antagonist and that the prostate GnRHR may differ from that present in pituitary. This finding could also complementarily explain the clinical results obtained with degarelix, leuprolide, and goserelin, showing differences in the efficacy and disease time free for PCa.

We conducted a global gene analysis in the BPH-1 prostate cell line to identify early gene changes that may indicate degarelix-responsive pathways. Our analysis showed that the effects of degarelix at 6h were minimal. These differences in gene expression, although small, were found to be statistically significant due to the low random variability in the samples. While the discrete changes may be due to the short treatment time, another possibility is that the efficacy of degarelix is cell cycle-dependent, and thus, early changes in gene expression were diluted by non-affected cell populations. Despite the few changes detected, we identified biological processes related to apoptosis and cell death, which were affected soon after degarelix treatment. This, together with the MTT data, showed a decreased viability at 24h, suggesting that degarelix acts rapidly to trigger apoptosis in a subpopulation of BPH-1 cells. The data also suggest that the initial effects of degarelix were mediated in part by a non-genomic mechanism.

Interestingly, we found a few genes affected at 6 and 24h, and instead, we identified that the biological processes related to cell adhesion, the cell cycle, and transcription were affected at 24h. GnRHR is known to be a Gi-coupled protein, and it is also possible that cross-talks with the growth factor receptors resulted in protein and lipid changes that altered the growth rate of BPH-1 cells [41,42,54,55]. Specifically, the gene-grouping analysis showed that, in addition to affecting the G-couple receptor related genes, degarelix-deregulated gene clusters, which included blood vessel development, apoptosis, cell proliferation, inflammation, and EGF-like genes. These affected processes could be of interest for BPH and PCa. It is intriguing that degarelix deregulated these genes; however, in the context of BPH, the sum of these gene changes could result in tissue remodeling, which could have an impact on prostate size. Taken together, the data suggest that degarelix has rapid non-genomic effects, followed by altered gene expression of clusters of genes known to result in tissue remodeling. Whether the pathways identified in the BPH-1 cell line translate to the clinic remains to be studied. However, the identified pathways are known to be involved in controlling the progression of BPH [56–58].

In addition, we found that the deregulation of genes related to the MAPK cascade. The MAPK signaling cascade is one of the major networks of interacting proteins that regulates cell growth and differentiation [59–62]. It involves distinct and specific downstream mediators that could be related to the decreased cell viability observed. Also, degarelix could deregulate MAPK directly through GnRHR or indirectly through the counteraction of growth factors and hormones present on BPH. Each MAPK cascade is activated by a specific stimulus, although a cross-talk between GnRH and growth factor signaling is also possible [41,42,54,55]. Complementary, steroidogenic, and cancerous tissues highly express translocator protein (TSPO), and its expression can be controlled by the following pathway component: PKC-MEK-ERK [63]. In fact, TSPO has been associated with various tissue- and disease-specific functions, and in our context, this component could be used by GnRHR intracellular signaling.

Previous studies conducted with a rat model of BPH showed differences in prostate gene expression after 42 days of exposure to cetrorelix-; these differences were found to be more related to inflammatory cytokines and growth factors [53]. While it is possible, it is unknown if this is the case for degarelix and prostate cells, since our main interest in this work is to identify the starting points or early molecular events triggered by degarelix; to determine this, we used 6 and 24h of exposure of cells to this antagonist and conducted a global gene expression study.

MALDI MS is an innovative tool to rapidly investigate the molecular composition of biological systems. A major benefit of MALDI MS is the capability to determine the distribution of hundreds of unknown peptides, lipids, or compounds in a single measurement [64,65]. Chemical stains, immunohistochemical tags, and radiolabels are common methods used to monitor molecular targets, but there are limits to the specificity and to the number of targets that can be monitored in a single measurement. MALDI MS can analyze complex mixtures ranging from small drug compounds to very large proteins, whether endogenous or exogenous. This technique requires very little sampling, and there is no need for the initial knowledge of target species. MALDI MS is a fast and easy approach to rapidly profile and characterize the molecular content (peptides, proteins, metabolites, drugs) of cultured cells [66–68]. This technique is therefore of great interest for biological, biomedical, and clinical studies, as well as for drug discovery and development. MALDI MS could provide the profiles to characterize normal, hyperplasia, and cancer cells, as well as to identify different protein and lipid mass spectra profiles as a fingerprint for degarelix-treated human prostate cells.

Using MALDI MS, we identified specific changes in m/z signal intensities in prostate cells induced by a GnRH antagonist (degarelix). Proteins and lipids were identified and found to be either up- or downregulated. The changes were robust enough to create a discriminatory profile induced by degarelix treatment. Interestingly, a more distinguished protein and lipid profile in WPE1-NA22 cells was found for currently unknown reasons.—These data further demonstrate that degarelix directly targets prostate cells, and this allowed us to obtain the first leads on the biological processes regulated by degarelix.

In conclusion, we showed that different types of human prostate cell lines (normal, hyperplasia, and cancer) are sensitive to the antiproliferative effect of degarelix, a GnRHR antagonist. Prostate cell growth was directly inhibited by degarelix, possibly involving a cell cycle-related mechanism and leading to apoptosis. Gene array results indicate a few interesting early molecular changes induced by degarelix that could have an impact in the prostate context, mainly controlling BPH growth. A MALDI analysis provided the basis to discriminate between the specific proteins and lipids found following degarelix treatment. Taken together, these findings suggest that GnRHR signaling within the prostate environment should be taken into consideration when designing therapies for the treatment of prostate diseases.

## Supporting Information

S1 Fig Two-dimensional (2D) density plots. (DOCX)

S2 Fig Degarelix spectrum. (DOCX)

S3 Fig MTT assay showing viability of BPH-1 cells pre-treated with either leuprolide or degarelix. (A-C), BPH-1 cells pre-treated with leuprolide (10μM) before treatment with increasing concentrations of degarelix. (D-F) BPH-1 cells pre-treated with degarelix (10μM) before the addition of increasing concentrations of leuprolide. Cells were analyzed 24, 48 and 72h after last treatment as indicated in each graph. Results shown are means from 3 independent experiments performed in triplicates.
(DOCX)

S1 Table Primers used for TaqMan Q-PCR analysis. (DOCX)

S2 Table Primers used for SYBR green Q-PCR analysis. (DOCX)

S3 Table Clusters of genes de-regulated by degarelix. (DOCX)
