# Supplementary figures and images for: In Search of the Molecular Mechanisms Mediating the Inhibitory Effect of the GnRH Antagonist Degarelix on Human Prostate Cell Growth
Source: PLoS One. 2015 Mar 26;10(3):e0120670. doi: 10.1371/journal.pone.0120670 (PMC4374753; doi:10.1371/journal.pone.0120670)

**S1 Fig.** **Two-dimensional (2D) density plots.**

**
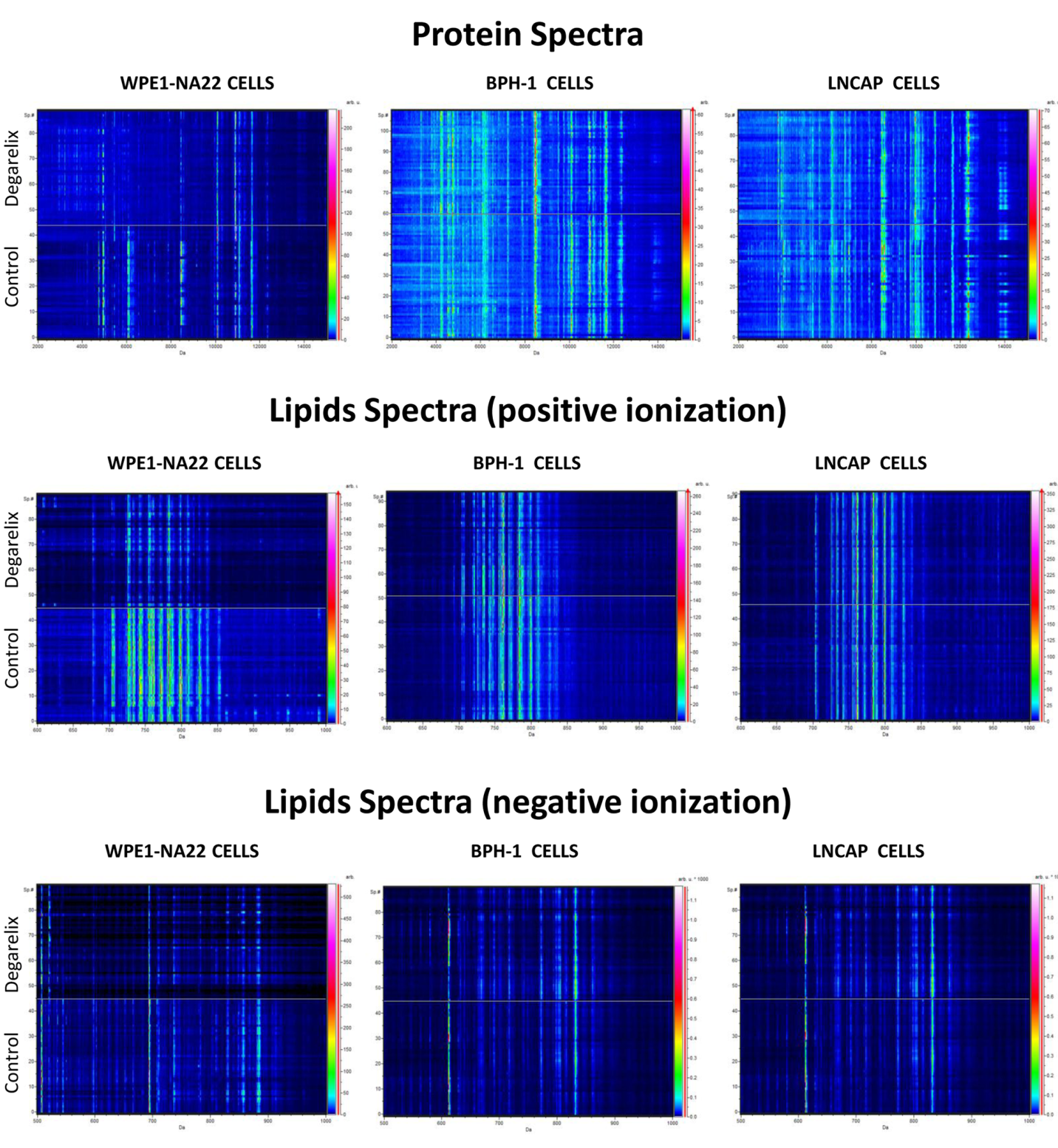
**

Supplement: S1 Fig — (DOCX) [file pone.0120670.s001.docx]

**S2 Fig. Degarelix spectrum.**

**
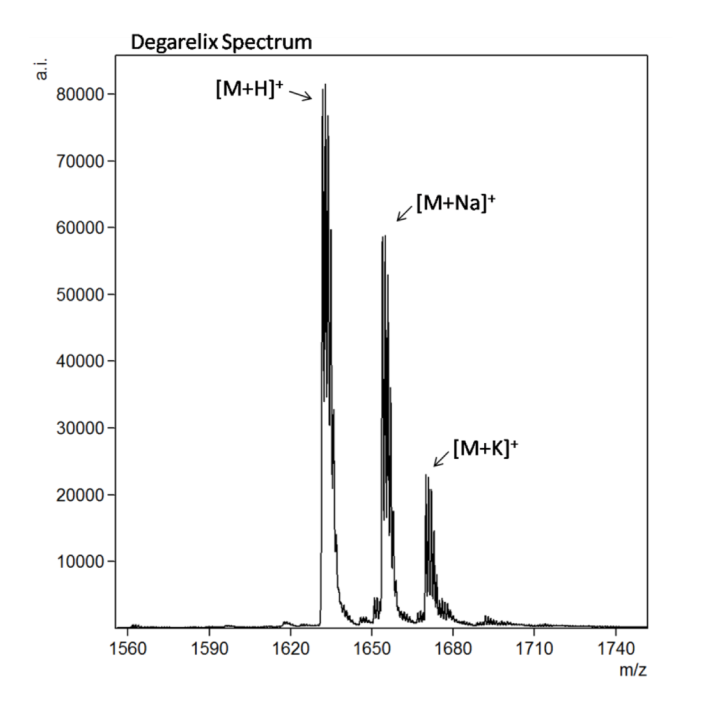
**

Supplement: S2 Fig — (DOCX) [file pone.0120670.s002.docx]
