# Supplementary material for: In Search of the Molecular Mechanisms Mediating the Inhibitory Effect of the GnRH Antagonist Degarelix on Human Prostate Cell Growth
Source: PLoS One. 2015 Mar 26;10(3):e0120670. doi: 10.1371/journal.pone.0120670 (PMC4374753; doi:10.1371/journal.pone.0120670)
Supplement: S3 Fig — (A-C), BPH-1 cells pre-treated with leuprolide (10μM) before treatment with increasing concentrations of degarelix. (D-F) BPH-1 cells pre-treated with degarelix (10μM) before the addition of increasing concentrations of leuprolide. Cells were analyzed 24, 48 and 72h after last treatment as indicated in each graph. Results shown are means from 3 independent experiments performed in triplicates. (DOCX) [file pone.0120670.s003.docx]

**S3 Fig. MTT assay showing viability of BPH-1 cells pre-treated with either leuprolide or degarelix.** (A-C), BPH-1 cells pre-treated with leuprolide (10µM) before treatment with increasing concentrations of degarelix. (D-F) BPH-1 cells pre-treated with degarelix (10µM) before the addition of increasing concentrations of leuprolide. Cells were analyzed 24, 48 and 72h after last treatment as indicated in each graph. Results shown are means from 3 independent experiments performed in triplicates.

**
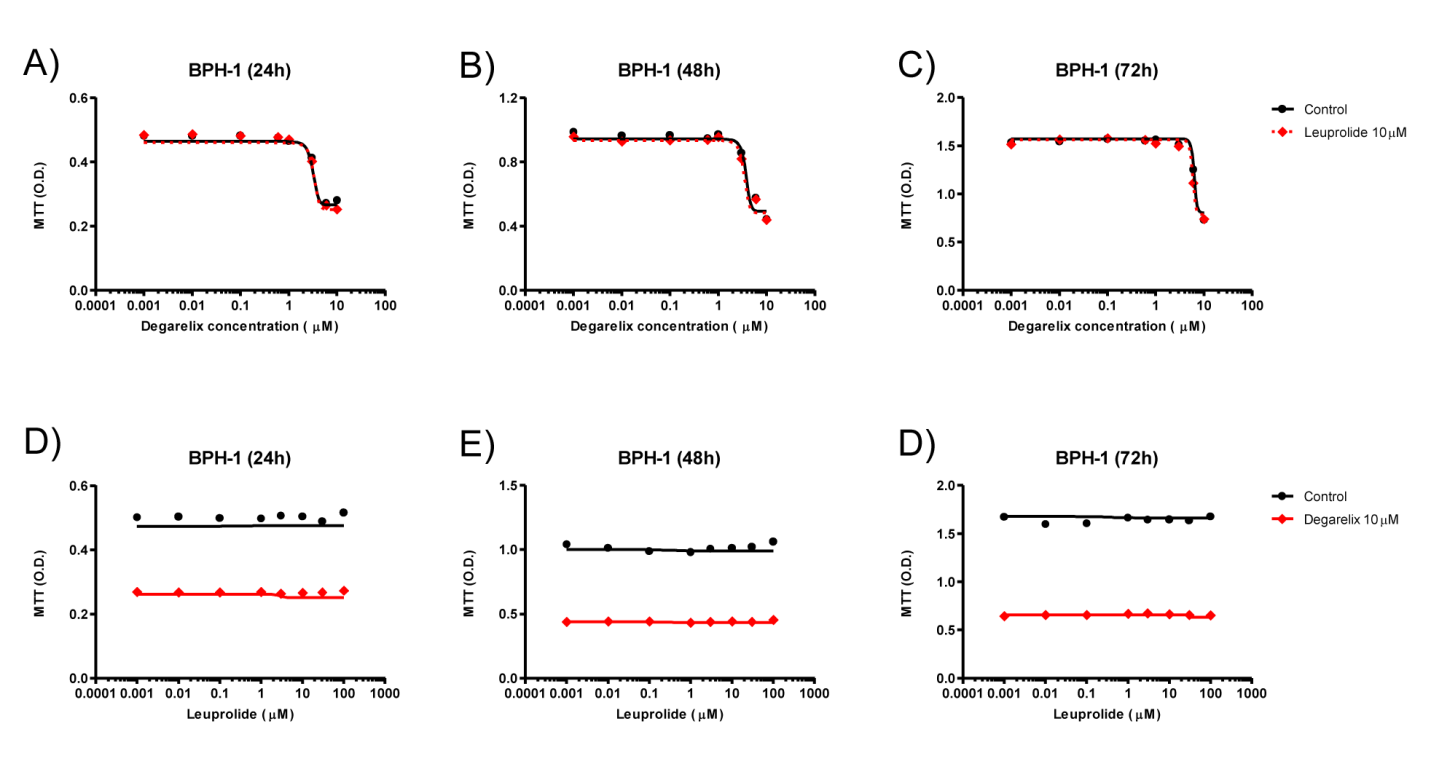
**
