# Supplementary material for: In Search of the Molecular Mechanisms Mediating the Inhibitory Effect of the GnRH Antagonist Degarelix on Human Prostate Cell Growth
Source: PLoS One. 2015 Mar 26;10(3):e0120670. doi: 10.1371/journal.pone.0120670 (PMC4374753; doi:10.1371/journal.pone.0120670)
Supplement: S1 Table — (DOCX) [file pone.0120670.s004.docx]

**S1 Table. Primers used for TaqMan® Q-PCR analysis**

| **Gene symbol** | **Ref Sequence** | **TacMan® reference** |
| --- | --- | --- |
| *GNRH R2* | AY233133 | Hs00369692_m1 |
| *GNRH1* | NM_001083111.1 | Hs00171272_m1 |
| *GNRH2* | NM_178331.1 | Hs01122823_m1 |
| *GAPDH* | NM_002046.3 | 4326317E |
