# Supplementary material for: In Search of the Molecular Mechanisms Mediating the Inhibitory Effect of the GnRH Antagonist Degarelix on Human Prostate Cell Growth
Source: PLoS One. 2015 Mar 26;10(3):e0120670. doi: 10.1371/journal.pone.0120670 (PMC4374753; doi:10.1371/journal.pone.0120670)
Supplement: S2 Table — (DOCX) [file pone.0120670.s005.docx]

**S2 Table. Primers used for SYBR green® Q-PCR analysis**

| **Gene symbol** | **Sequence** |
| --- | --- |
| *GNRHR1* | F CCAAGAACAATATACCAAGAGCACGG |
|  | R CCAGCAGACAGTAAATGAAGTGGCAA |
