# Supplementary material for: In Search of the Molecular Mechanisms Mediating the Inhibitory Effect of the GnRH Antagonist Degarelix on Human Prostate Cell Growth
Source: PLoS One. 2015 Mar 26;10(3):e0120670. doi: 10.1371/journal.pone.0120670 (PMC4374753; doi:10.1371/journal.pone.0120670)
Supplement: S3 Table — (DOCX) [file pone.0120670.s006.docx]

**S3 Table. Clusters of genes de-regulated by degarelix**

| **Cluster 1: Genes related to blood vessels development** | | |
| --- | --- | --- |
| *CD44* | CD44 molecule (Indian blood group) | |
| *PROX1* | prospero homeobox 1 | |
| *NTRK2* | neurotrophic tyrosine kinase, receptor, type 2 | |
| *WARS2* | tryptophanyl tRNA synthetase 2, mitochondrial | |
| *SMARCA4* | SWI/SNF related, matrix associated, actin dependent regulator of chromatin, subfamily a, member 4 | |
| *PRRX1* | paired related homeobox 1 | |
| *ZMIZ1* | zinc finger, MIZ-type containing 1 | |
| *CDH13* | cadherin 13, H-cadherin (heart) | |
| *FGF9* | fibroblast growth factor 9 (glia-activating factor) | |
| *FLT1* | fms-related tyrosine kinase 1 (vascular endothelial growth factor/vascular permeability factor receptor) | |
| *SEMA5A* | sema domain, seven thrombospondin repeats (type 1 and type 1-like), transmembrane domain (TM) and short cytoplasmic domain, (semaphorin) 5A | |
| *ROBO2* | roundabout, axon guidance receptor, homolog 1 (Drosophila); similar to roundabout 1 isoform b | |
| *CTNNB1* | catenin (cadherin-associated protein), beta 1, 88kDa | |
| *SRF* | serum response factor (c-fos serum response element-binding transcription factor) | |
|  |  | |
| **Cluster 2: Genes related to the MAPK pathway** | | |
| *NTRK2* | neurotrophic tyrosine kinase, receptor, type 2 | |
| *MAP4K5* | mitogen-activated protein kinase kinase kinase kinase 5 | |
| *DHRS2* | dehydrogenase/reductase (SDR family) member 2 | |
| *MLL* | myeloid/lymphoid or mixed-lineage leukemia (trithorax homolog, Drosophila) | |
| *CHST11* | carbohydrate (chondroitin 4) sulfotransferase 11 | |
| *ETS1* | v-ets erythroblastosis virus E26 oncogene homolog 1 (avian) | |
| *CTLA4* | cytotoxic T-lymphocyte-associated protein 4 | |
| *CTTNBP2* | cortactin binding protein 2 | |
| *ESR1* | estrogen receptor 1 | |
| *PROX1* | prospero homeobox 1 | |
| *FNTA* | farnesyltransferase, CAAX box, alpha | |
| *ZMIZ1* | zinc finger, MIZ-type containing 1 | |
| *THPO* | thrombopoietin | |
| *CDK6* | cyclin-dependent kinase 6 | |
| *ADRB3* | adrenergic, beta-3-, receptor | |
| *BMPR1A* | bone morphogenetic protein receptor, type IA; similar to ALK-3 | |
| *FLT1* | fms-related tyrosine kinase 1 (vascular endothelial growth factor/vascular permeability factor receptor) | |
| *PRRX1* | paired related homeobox 1 | |
| *CDH13* | cadherin 13, H-cadherin (heart) | |
| *CTNNB1* | catenin (cadherin-associated protein), beta 1, 88kDa | |
| *FGF9* | fibroblast growth factor 9 (glia-activating factor) | |
| *HIPK2* | homeodomain interacting protein kinase 2; similar to homeodomain interacting protein kinase 2 | |
|  |  | |
| **Cluster 3: Genes related to apoptosis** | | |
| *FXR1* | fragile X mental retardation, autosomal homolog 1 | |
| *DNASE1* | deoxyribonuclease I | |
| *CTNNB1* | catenin (cadherin-associated protein), beta 1, 88kDa | |
| *MLL* | myeloid/lymphoid or mixed-lineage leukemia (trithorax homolog, Drosophila) | |
| *USH1C* | unc-5 homolog B (C. elegans) | |
| *HIPK2* | homeodomain interacting protein kinase 2; similar to homeodomain interacting protein kinase 2 | |
| *APP* | amyloid beta (A4) precursor protein | |
| *TRIM69* | tripartite motif-containing 69 | |
|  |  | |
| **Cluster 4: Genes related to G-protein receptors** | | |
| *BRD8* | bromodomain containing 8 | |
| *FLT1* | fms-related tyrosine kinase 1 (vascular endothelial growth factor/vascular permeability factor receptor) | |
| *APP* | amyloid beta (A4) precursor protein | |
| *ERBB2IP* | erbb2 interacting protein | |
| *PTPRG* | protein tyrosine phosphatase, receptor type, G | |
| *CTNNB1* | catenin (cadherin-associated protein), beta 1, 88kDa | |
| *CD59* | CD59 molecule, complement regulatory protein | |
| *NTRK3* | neurotrophic tyrosine kinase, receptor, type 3 | |
| *FGF9* | fibroblast growth factor 9 (glia-activating factor) | |
| *FNTA* | farnesyltransferase, CAAX box, alpha | |
| *NTRK2* | neurotrophic tyrosine kinase, receptor, type 2 | |
| *MACF1* | microtubule-actin crosslinking factor 1 | |
| *HIPK2* | homeodomain interacting protein kinase 2; similar to homeodomain interacting protein kinase 2 | |
| *INVS* | inversin | |
| *SFRP2* | secreted frizzled-related protein 2 | |
| *BMPR1A* | bone morphogenetic protein receptor, type IA; similar to ALK-3 | |
| *ZFYVE16* | zinc finger, FYVE domain containing 16 | |
| *RGS18* | regulator of G-protein signaling 18 | |
| *GNB1* | guanine nucleotide binding protein (G protein), beta polypeptide 1 | |
| *GNG12* | guanine nucleotide binding protein (G protein), gamma 12 | |
| *GRM7* | glutamate receptor, metabotropic 7 | |
| *GPR101* | G protein-coupled receptor 101 | |
| *LGR4* | leucine-rich repeat-containing G protein-coupled receptor 4 | |
| *RGR* | retinal G protein coupled receptor | |
| *FPR2* | formyl peptide receptor 2 | |
| *OR5L2* | olfactory receptor, family 5, subfamily L, member 2 | |
| *ADRB3* | adrenergic, beta-3-, receptor | |
| *OR13C4* | olfactory receptor, family 13, subfamily C, member 4 | |
|  |  | |
| **Cluster 5: Genes related to inflammatory response** | | |
| *F11* | coagulation factor XI | |
| *SRF* | serum response factor (c-fos serum response element-binding transcription factor) | |
| *CD59* | CD59 molecule, complement regulatory protein | |
| *GRM7* | glutamate receptor, metabotropic 7 | |
| *HLA-C* | major histocompatibility complex, class I, C; major histocompatibility complex, class I, B | |
| *CD84* | CD84 molecule | |
| *NFX1* | nuclear transcription factor, X-box binding 1 | |
| *FPR2* | formyl peptide receptor 2 | |
| *IL23A* | interleukin 23, alpha subunit p19 | |
| *CD44* | CD44 molecule (Indian blood group) | |
|  |  | |
| **Cluster 6: Genes related to EGF-like proteins** | | |
| *MUC13* | | mucin 13, cell surface associated |
| *LTBP4* | | latent transforming growth factor beta binding protein 4 |
| *GSPG5* | | chondroitin sulfate proteoglycan 5 (neuroglycan C) |
